# Surgical Smoke and Airborne Microbial Contamination in Operating Theatres: Influence of Ventilation and Surgical Phases

**DOI:** 10.3390/ijerph17155395

**Published:** 2020-07-27

**Authors:** Francesco Romano, Samanta Milani, Jan Gustén, Cesare Maria Joppolo

**Affiliations:** 1 Dipartimento di Energia, Politecnico di Milano, 20125 Milan, Italy; samanta.milani@polimi.it (S.M.); cesare.joppolo@polimi.it (C.M.J.); 2 Building Services Engineering, Chalmers University of Technology, SE-41296 Gothenburg, Sweden; contaminant.control.consultants@gmail.com

**Keywords:** operating theatres, ultrafine particle, airborne microbiological contamination, surgical smoke, ventilation system, surgical phases

## Abstract

Air cleanliness is a crucial factor in operating theatres (OTs), where the health of patients and staff must be preserved by controlling air contamination. Particular attention must be paid to ultrafine particles (UFPs) size range, generated for instance by electrosurgical instruments (ESTs). OT contamination is also affected by ventilation systems, medical staff and their gowning system, staff routines, instruments, etc. This comparative study is based on experimental measurements of airborne microbial contamination and UFPs carried out during real ongoing surgeries in two OTs equipped with upward displacement ventilation (UWD) and hybrid ventilation, with unidirectional airflow on the operating table and peripheral mixing (UDAF+Mixing) ventilation systems. Airborne contamination concentration at the exit grilles has been analyzed as function of four different surgical phases normally performed during an operation. Results highlight that airborne contamination is influenced by the activities carried out during the surgical phases. EST usage affects the contamination level more than staff size during operation observed. Colony forming unit (CFU) values in the protected area close to the patient’s wound are influenced more by the type of ventilation system than by surgical phases. CFU values decrease by 18 to 50 times from the UWD system to the hybrid one. The large airflow volumes supply together with high air velocities in OTs equipped with UDAF+Mixing systems guarantee a better and a safer airborne contamination control for patients and medical team in comparison with UWD systems.

## 1. Introduction

Airborne contamination control is a fundamental requirement in Operating Theatres (OT) environments to preserve patient and medical staff health. Efficient ventilation systems in healthcare facilities can maintain low airborne particle and microbial air contamination in the environment, reducing the chances of infections by airborne transmission and ensuring good and safe working conditions for medical staff. The novel coronavirus Covid-19 emergency has raised awareness of virus transmission to surgical team in the operating room and to outer premises [1,2]. The microbes carrying particles are generally considered the main source of contamination during surgeries within OT premises [3,4,5,6]. Other sources of contamination are medical staff, surgical routines, and patients.

Particle shedding from the medical team can be easily controlled wearing proper garments. The technical clothing used by medical staff protect them from external contamination and act as filtration barrier for the contamination generated [7,8,9]. The level of human activity, together with the gowning filtration performance, and the medical staff procedures may change the amount of total and biological airborne particles released by humans [5,9,10,11,12].

Moreover, the medical team and patients may also be affected by airborne contamination generated in the surrounding of their bodies, the so-called “personal cloud”, by endogenous and exogenous contamination sources [13,14,15].

Another significant airborne contamination source in OTs is the surgical smoke produced by electrosurgical tools (ESTs) during routine surgeries [16,17,18,19,20,21]. ESTs contribute to the production of ultrafine (UFP) and fine (FP) particles, with a size range from 0.007 µm to 0.42 µm [22]. Small airborne particles, such as UFP and FP, are inhalable and may settle in the deepest part of the respiratory tract, leading to negative health consequences [23,24].

ESTs generate particles which can easily spread, by convection and diffusion, far from the wound in a short time [25] and the concentration remains rather high even after ESTs usage [24,26]. The health risks associated with the transmission of diseases to medical staff in OTs via viral and bacterial pathogens in surgical smoke have been studied in several investigations [18,27,28,29,30,31,32,33,34]. To the best of our knowledge, at the moment there is no scientific evidence that surgical smoke may contain and vehicle the SARS Cov-2 virus [1,2,35].

Studies on the potential health risks due to the exposure to airborne and microbiological contaminants have been carried out focusing on the contamination doses in offices [36,37], public schools [38], and hazardous work environments [39]. 

Recently, the airborne particle emission rate and the contaminant doses received in an operating theatre by medical staff due to surgical smoke have been experimentally assessed [24,40], while a first numerical approach of UFP transport in OT equipped with turbulent diffusers has been carried out by Salahudeen et al. [41].

Many standards and guidelines address the issue of surgical smoke. Occupational Safety and Health Administration (OSHA) [42] states that nearly 500,000 healthcare workers are exposed to laser and electrosurgical smoke in OTs. OSHA and the National Institute for Occupational Safety and Health (NIOSH) [43] recommend the use of preventive measures and personal protective equipment (PPE) against surgical smoke, and the Association of periOperative Registered Nurses (AORN) [44], Association of Surgical Technologists (AST) [45], American National Standards Institute (ANSI) [46] and Liu [47] also suggest the use of local exhaust ventilation (LEV) system to protect workers from surgical smoke hazard. In addition, operating room team members are concerned about surgical smoke safety [48]. Nowadays, there are no specific UFP limit values set by OSHA standard for the control of surgical smoke plume generated by laser and ESTs [49] nor ventilation and filtration system requirements.

The most adopted ventilation systems in OT environments are: (i) unidirectional airflow (UDAF), (ii) mixing, and (iii) upward displacement systems (UWD). The UDAF ventilation system generates a low turbulence airflow directed from the high-efficiency ceiling filters toward the floor. In the mixing airflow ventilation, high induction air mixing diffusers supply clean air to dilute the concentration of airborne contaminants in the environment. In the UWD system, low and cold airflow volume is dislocated at floor level by low turbulence air diffusers while the extraction grills are generally located in the ceiling. 

In recent years, the complexity of surgical operations has required a multidisciplinary approach in which medical diagnostic equipment is inside OTs, creating a hybrid between a common OT and a diagnostic room, the so-called Hybrid OT. Hybrid OTs can simultaneously use different ventilation principles: a UDAF system in the protected zone around the surgical table and a mixing system in the surrounding areas.

The efficiency of these ventilation principles in terms of contamination control and reduction of Surgical Site Infections (SSIs) has been questioned in several works paying attention only to the health risks for the patient [4,5,50,51,52,53]. Few works have studied the influence of the ventilation system on the health risks for the medical team due to surgical smoke [16,17,24,54].

OT ventilation systems are generally equipped with terminal filters of two types: high efficiency particulate air (HEPA) or ultra low particulate air (ULPA). These filters process the total airflow rate supplied in the OT. Both HEPA and ULPA filters have very high particle retention efficiency for the most penetrating particulate size (MPPS), whose dimension is larger than the contamination produced by ESTs within OTs. Therefore, UFPs and gaseous components may be reintroduced into the OT environment by the recirculated airflow [55].

To quantify and to compare the airborne contamination in OTs during real surgeries, an experimental campaign has been conducted, evaluating the amount of UFPs and airborne microbial contamination during different surgical phases. In addition, to assess the influence of the air ventilation systems on the total airborne contamination spread within OTs, we monitored two OTs equipped with different systems, geometries, and layouts during real surgical activities. A further aim of this study is to enrich the scientific literature with new experimental results on UFPs and microbial contamination generated by surgical smoke in real surgeries. To the best of our knowledge, this is one of the first studies offering a comprehensive investigation of the exposition level of medical teams to airborne contamination in real surgeries comparing OTs with different ventilation systems. The results of this comparative study are potentially helpful in choosing the right ventilation system to reduce the surgical smoke and airborne microbial contamination generated in OT environments during the surgical phases of real surgeries. This body of evidence may contribute to limit the airborne particle and microbial contamination, and consequently the risk of health hazards for patients and for surgical teams.

## 2. Materials and Methods 

In the comparative study proposed, an experimental campaign was conducted during 13 real surgical operations in two operating theatres equipped with different ventilation systems. Ultrafine particle measurements have been carried out over the entire surgery duration, while microbiological measurements have been collected during specific stages of surgery.

### 2.1. Operating Theatres

**Figure 1:**
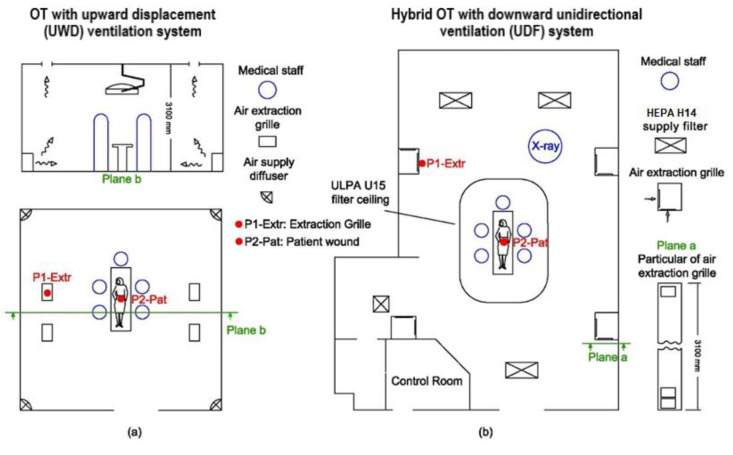
Operating theatres layout and sampling probe position: (**a**) OT1 with UWD; (**b**) OT2, Hybrid OT (UDAF+Mixing).

The experimental measurements have been carried out during real surgical operations in two operating theatres in different Swedish Hospitals. A brief presentation of the two OTs’ technical characteristics is given in the following:

1. OT1 is an upward displacement airflow system (UWD) OT, equipped with four HEPA H14 filter air supply diffusers located in the corners of the room at floor level. The extraction grilles are positioned on the ceiling around the surgical table, at the center of the room (Figure 1a). The supply airflow rate is 0.56 m^3^/s and the extracted airflow rate is 0.41 m^3^/s.

2. OT2, is a Hybrid OT with UDAF system. It consists of a clean zone above the surgical table supplied with a downward unidirectional vertical airflow. The airflow rate is 3.6 m^3^/s, it is introduced by a ULPA U15 ceiling filter with an area of 12.4 m^2^. In the peripheral area, outside UDAF zone, a mixing ventilation system supplies an airflow rate of 0.7 m^3^/s through three HEPA H14 filter diffusers which dilute the airborne contaminants. Four extraction columns are located on the side walls of the OT which extract an airflow rate of 4.2 m^3^/s (see Figure 1b). The average air changes per hour (ACH) are 57, although in the clean area are higher than the peripheral area.

Dimensions and technical characteristics of the evaluated OTs are shown in Table 1.

Both OTs operate with 70% recirculating air and 30% external supply air. A 10 Pa overpressure compared to adjacent rooms ensures air exfiltration from the OTs. The thermo-hygrometric indoor air conditions were kept constant at 20 °C and 55% relative humidity (RH). Values are referred to the air extraction plenum.

### 2.2. Instruments and Probe Positioning

Ultrafine particle concentration has been monitored using ultrafine particle counters (UFP-C, P-Trak mod. 8825, TSI Inc., Shoreview, MN, USA). The instruments measure particles in the range 0.02 to 1 µm, within a detection limit of 0 to 5 × 10^5^ pp/cm^3^. According to standard ISO 27891 [56], the air sampling flow rate is 0.1 L/min. The measures were carried out with a log interval of 10 s without any delay between consecutive records. Sampling location has been chosen after a sensitivity analysis of the contamination values in preliminary tests. The sampling probe was positioned near the air extraction grille (P1-extr), a point representative of the entire UFP contamination of the OT outside the operating table (see Figure 1).

Airborne aerobic microorganisms were measured with 10 min samples by a MD-8 Sartorius air sampler (Sartorius Stedim Biotech GmbH, Goettingen, Germany) at a constant airflow rate of 100 L/min with accuracy of ±5% of the reading. The filter holder was equipped with a sterile gelatine filter (diameter 80 mm, pore size 0.3 µm). After sampling, the gelatine filter was placed on a Petri dish and incubated for 48 h at 36 °C and then exposed for 48 h at room temperature. The culture media was a non-selective Columbia agar with 5% horse blood.

The sampling of microbiological air contamination was performed during each surgery phase. The air sample probe was located within the surgical site as close as possible to the patient, 30 cm from the wound (named point P2-pat in Figure 1) Measurements were carried out by a scrub nurse.

### 2.3. Experimental Procedure and the Role of Medical Staff

UFPs and airborne microbiological contamination in operating theatres during real surgery are sensitive to many conditions as ventilation system, cleaning procedures, personnel, type of tissue cut by ESTs, surgical procedures, etc. Surgical procedures and movements have been performed in a routine, standardized manner, although they are difficult to plan and to perform on a recurring basis, as indicated in previous works [3,4,13].

In collaboration with the medical staff, a surgical operation was defined and divided into four phases, as described in the following.

1. Preparation:
  (i) Preparation of the room and the equipment. Equipment and tools are kept sterile passing from the grey outside area to the sterile area (protected zone) and covered with sterile plastic layers. (ii) Patient entry and anesthesia procedure. This step takes place before or after the patient is transferred to the operating table, depending on the type of surgical operation. In the preparation for the operation, the body is shaved, cleaned, disinfected, and covered to expose only the portion to be treated. In this phase the door is opened frequently and the number of personnel in the room is variable, from 2–3 nurses during the preparation of the equipment to a higher number of nurses and anesthetists in the subsequent moments.
2. Body opening
  Patient body is opened, and tissues cut by ESTs as preparatory step for the main surgical phase. This part may take from few minutes to hours, depending on the type of operation. The number of people varies from one or two surgeons, and one or two anesthetists, plus a variable number of nurses.
3. Main surgery
  The surgery phase is performed. The use of EST is limited compared to the previous phase. The size of medical staff is generally constant, and the door opening is minimized.
4. Body closing
  The patient’s wound is closed by surgeons. ESTs usage is limited and mainly dedicated to cauterization of tissues and small vessels. The duration of this phase is usually short, although it may take hours in the case of orthopedic operations.

After the fourth phase, the operation is completed and the patient leaves the operating theatre. At the end of each operation, the room is cleaned and prepared for a new operation.

Medical staff involved in the research activities were duly instructed to perform as many pre-established movements and procedures as possible during real surgeries. The medical staff wears technical clothing consisting of blouse and trousers (50% polyester and 50% cotton), common hospital shoes, head cover, and protective vest for radiation equipment. In orthopedic and liver resection operations only, technical clothing is sterile and composed of blouse and trousers (30% nylon and 70% cotton), common hospital shoes, single use helmet. In addition, surgical smock, mask and double pair of sterile gloves are used in all operations.

UFPs and airborne microbiological contaminations were measured according to the detailed aforementioned specifications. Before every sampling beginning, sampling probe, instruments, and cables were cleaned and disinfected, and a zero count was made by all air samplers. In addition, during surgeries, staff size and door openings were monitored.

### 2.4. Ethics

Swedish legislation (Act 2003:460, Amended SFS 2008:192) does not demand ethical permission for this type of observational studies that do not involve patients. However, informed consent in line with the Declaration of Helsinki was given to all OT teams (World Medical Association, 2013). The medical person in charge from The University Hospital was involved in the research work.

## 3. Results and Discussion

Experimental measurements during real surgeries were carried out with the aim of evaluating the airborne contamination load generated in OTs equipped with different ventilation schemes, UWD (OT1) and UDAF (OT2). Table 2 shows the main experimental data obtained by the surgeries monitored in the investigated OTs. Averaged data on similar surgeries are reported.

The types of surgeries monitored were: neurological (callosotomy), orthopedic, endo-vascular-aortic repair (EVAR), liver resection, and nasal cavity cancer removal.

Even though the operations were very different, the number of medical personnel and UFP concentrations observed in the extracted air (P1-extr) were of the same order of magnitude between different operations carried out in the same OT, with some exceptions. A significative difference arose for the UFPs concentration during orthopedic surgeries carried out in OTs with different ventilation systems, with 940.1 vs. 18.1 pp/cm^3^, respectively in OT1 and OT2 (Table 2). The UFP concentration in the OTs seems to be more related to the type of activity carried out and to the type of ventilation system rather than to the number of people involved. In particular, the highest number of people was observed in the cancer removal operation in OT2, where 10 staff members (two surgeons, six nurses, two anesthetists) plus the patient are present. In this case, the UFPs concentration values were consistent with the ones experienced in EVAR surgery carried out in OT2. The low value of UFPs contamination in those surgeries was the result of the routine activities applied by staff which do not require high vigor and intensity, with a consequent low human particle emission rate, in addition to the low ESTs’ frequency usage. The lowest number of people, on the other hand, was observed during orthopedic procedures (two surgeons, three nurses, one anesthetist). During these operations, the contamination concentration values recorded in both operating theatres were among the highest due to the extensive surgical tools usage and activities generally needed for those surgeries.

The door opening frequency seems to have no significant influence on ultrafine and microbiological particle concentrations: this agrees with the work carried out by Montagna et al. [57] but is in contrast to other studies [3,4,5,20]. Moreover, the UFP and microbiological contamination showed differences between the values recorded in the two OTs evaluated. Airborne microbial contamination in OT1 was on average 20 CFU/m^3^, while was near zero in surgeries carried out in OT2. In this case, an influence of the number of people on airborne microbial contamination in operating theatres with low ACH might be observed, consistent with other studies [4,20,49]. In this case, a fundamental role is assigned to the air velocity intensity and the air path direction given by the ceiling filter in presence of considerable ACH values; the higher the ACH, the higher the velocity and the better the sweep effect by the unidirectional downward airflow.

The study also investigated the influence that the four surgical phases had on the airborne concentration during real surgeries. Table 3 shows the number of people attending each phase, time averaged over the surgical operations performed.

**Figure 2:**
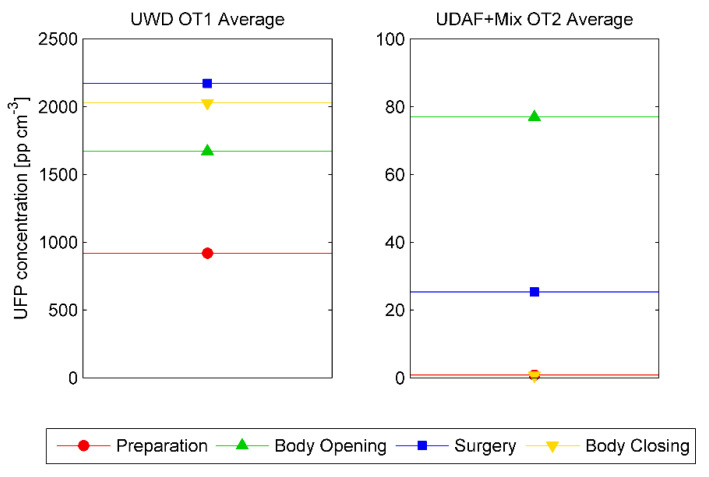
Time average UFPs concentration for UWD OT1 (left) and Hybrid OT2 (right) in all surgeries as function of surgery phases.

The subdivision of the surgery operation in four phases is shown in Figure 2 in regards to the time average UFPs concentration at the extraction grille (P1-extr) in the two OTs for all the surgeries investigated.

UFP concentrations produced in the Hybrid OT (OT2) was close to 20 times lower than the one generated in OT1, equipped with the UWD system. Although a small increase in the presence of medical staff during the body opening and the operation phases, this is not likely to explain such a large difference in UFPs concentrations. In OT2, the highest values were reached during the surgery and the body opening phases, while in OT1, UFPs values were stable and high throughout all the phases. In the latter case, the poor airflow ventilation is unable to significantly decrease the contamination level in the OT environment if compared with the high ACH and with the high air velocities present in the OTs equipped with UDAF+Mixing systems.

**Figure 3:**
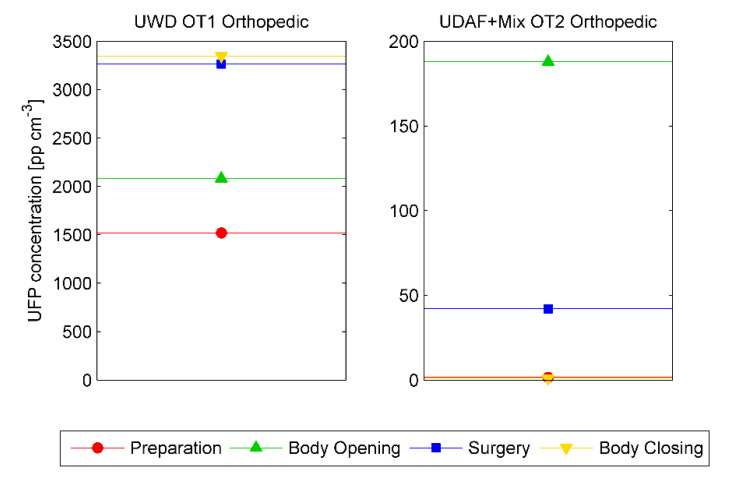
Time average UFPs concentration for UWD OT1 (left) and Hybrid OT2 (right) in orthopedic surgeries as function of surgery phases.

Same conclusions can be obtained by comparing the UFPs contamination level generated by the same type of surgical operation when carried out in OTs with different ventilation systems, as shown in Figure 3. In the orthopedic operation performed in OT1 (with UWD dislocation system) the results were 10 to 50 times higher than in OT2 (hybrid room), depending on the selected phase.

Regardless of the ventilation system adopted by the OTs, the phases with the highest concentration (“phase 2—body opening” and “phase 3—surgery”) were closely related to the use of ESTs, as described by Romano et al. [17]. 

As 70% of the air supplied in the OTs is filtered and recirculated in local air handling unit (AHU), a higher indoor concentration of UFPs may also correspond to a worse filtration stage for the recirculated air. In this study, OT1 was equipped with HEPA H14 filters while Hybrid OT2 had ULPA U15 filters. The large difference in airflow rate among the two OTs under evaluation, and partly the filters installed, can explain why the last surgical phases in OT1, in comparison with the values obtained in OT2, achieved consistently higher contamination values.

**Figure 4:**
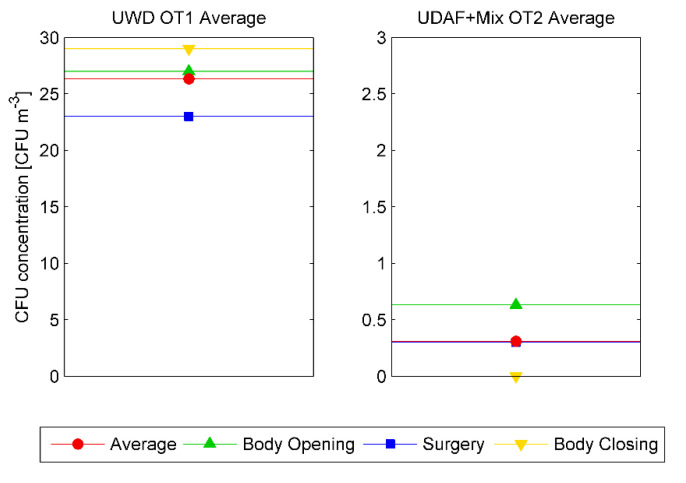
Time average CFU/m^3^ values for UWD OT1 (left) and Hybrid OT2 (right) in all surgeries as function of surgery phases.

An evaluation of the viable particle concentration near the surgical wound (Point P2-Pat) during the surgery phases is shown in Figure 4, averaged over the duration of all the surgical operations monitored. It is remarked that the CFU concentration was less than 1 CFU/m^3^ during all phases in Hybrid OT2, while in OT1 it was quite high with an average value of 27 CFU/m^3^.

**Figure 5:**
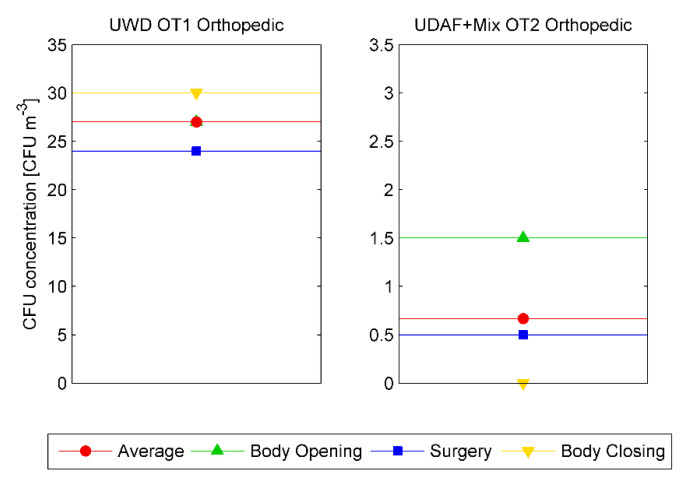
Time average CFU/m^3^ values for UWD OT1 (left) and Hybrid OT2 (right) in orthopedic surgeries as function of surgery phases.

It’s noteworthy that phases where people are required to perform intensive activities have the highest microbiological emission rate, and therefore CFUs emission. Differences in performance between the ventilation systems adopted by the two OTs are emphasized by the airborne microbiological contamination even more than UFPs values. In this case, the comparative analysis of the microbial contamination during orthopedic surgeries performed in the two OTs, as shown in Figure 5, confirms the same contamination trend as for the UFPs concentrations, with very low CFU values for Hybrid OT ventilation systems.

The Hybrid OT with UDAF+Mixing system can keep the airborne CFUs values in the protected area around the operating table far below those in OT1 with UWD system, in detail from 18 to more than 50 times less. The best performance of the Hybrid OT is directly related to UDAF system, which processes a large airflow rate. Large ACHs means high air velocity intensity. The latter two parameters in a UDAF ventilation system contribute in displacing and removing airborne contamination more rapidly from the protected area where medical personnel remain during surgeries, maintaining a low level of airborne contaminants, and thus reducing the health risk for patients and medical teams. However, the benefits offered by the ventilation system of the Hybrid OT are counter balanced by the high energy consumption for ventilation.

## 4. Limitations

The proposed study made a comparison of the results obtained in different OTs and surgeries. The human physiological characteristics (age, gender, race, activities), the environmental conditions (air velocity, air diffusion, temperature, and relative humidity), and the type of surgeries may set limitations of this experimental study. The limited number of real operations monitored, and the activities performed by the medical teams, do not always follow the measurement protocol due to medical urgency, are other minor limitations of this work. The different filter sections in the two OTs, HEPA H14 in OT1 and ULPA U15 in OT2, can also add some minor limitations to this study, even though, for the particle size of interest in this study, differences among the two filter types can be considered minimal. The measurement protocol used guarantees the accuracy and the reproducibility of experimental measurements, so that data can be used as comparative values for judging the key performance parameter of an OT. In the future, a new extensive experimental campaign should be performed to discern in detail the effect of surgical phases, EST tools, ventilation systems, and surgical gowning systems on the total and on the microbiological contamination.

A more in-depth comprehensive medical knowledge of the correlation between exposure levels and human health related risks from surgical smoke would be highly desirable.

## 5. Conclusions

The ventilation system is a key factor in providing a clean and safe environment within OTs. This comparative study has confirmed that different ventilation schemes lead to different results in terms of UFPs and airborne microbiological cleanliness. This may consequently affect the choice and the quality of the health protection levels for medical staff and patients. This investigation dealt with monitoring the total and viable airborne contamination in different surgical phases in OTs equipped with two types of ventilations systems (OT1-UWD versus OT2-UDAF+Mixing).

Surgical smoke generation during ESTs usage increases the UFPs concentrations within the OTs regardless the type of ventilation system. However, differences in UFP concentration values arises as a function of the ventilation schemes. OTs equipped with UDAF or hybrid (UDAF+Mixing) systems can dilute and remove the airborne contamination from the source generation point better and faster. This outcome is generated by the large airflow rate supplied and by the airflow path. A stable and constant downward unidirectional sweep effect guarantees a safe protection barrier for medical staff and patients located within the protected zone delimited by the UDAF area. In contrast, the UWD ventilation pattern shows difficulties in keeping low contamination levels due to low airflow rates and upward displacement airflow paths.

The average microbiological concentrations during surgeries, and at each surgical phase, show the effectiveness of the UDAF ventilation scheme in keeping the area protected. The final airborne microbiological contamination in OTs with low airflow rates and incorrect air diffusion is affected by the surgical phase and the size of medical team, as occurred in UWD OT1. The door opening frequency weakly influences the airborne concentration in the observed surgeries.

From an engineering perspective, a local exhaust ventilation system (LEV) properly designed for ESTs tools would be effective in reducing the surgical smoke diffusion. A combination of a Hybrid (UDAF+Mixing) ventilation scheme and LEV system could ensure an adequate level of air cleanliness inside OTs when using ESTs. UFPs, as well as total and viable airborne contamination, can be easily reduced with effective surgical procedures, proper gowning system and medical team’s awareness on health hazard risks. Although OTs with hybrid ventilation schemes demand more energy consumption than other systems, the benefit in terms of airborne contamination control and reduced health risks is predominant.

## Figures and Tables

**Table 1:** Technical data of the evaluated OTs.

OT	Ventilation System	Area	Volume	Supply Airflow Rate	ACH	Surgical Lamps	Terminal Filters
		(m^2^)	(m^3^)	(m^3^/h)	(h^−1^)	(#)	(-)
OT1	UWD	36	108	2.016	19	2	H14 (UWD)
OT2	UDAF+Mixing	100	270	15.480	57	2	U15 (UDAF)+ H14 (Mixing)

**Table 2:** Parameters and results of the experimental measurements.

Test Parameters						
OT	OT1	OT1	OT2	OT2	OT2	OT2
Surgical Operation Type	Neurological	Orthopedic	EVAR	Liver Resection	Orthopedic	Cancer Removal
Number of Surgeries Monitored	1	3	4	1	3	1
Total Hours Recorded (h)	6	7	22	6	16	4
Mean Number of Personnel *	8	6	8	9	6	10
Door Open Frequency * (1/min)	0.17	0.24	0.36	0.32	0.24	0.17
Results						
UFP 0,02–1 µm * (pp/cm^3^) (min; max)	1.52 (0; 34)	940.1 (0; 9258)	0.16 (0; 65)	18.7 (0; 2392)	18.1 (0; 5280)	0.68 (0; 28)
CFU * (CFU/m^3^) (min; max)	23 (10; 34)	27 (6; 48)	0.4 (0; 1)	0.1 (0; 2)	0.7 (0; 2)	0 (0; 0)

* Averaged values for similar surgeries.

**Table 3:** Average medical staff presence during different phases of surgeries monitored.

Operation Phase	OT1	OT2
Preparation	6	6
Body opening	7	10
Surgery	7	8
Body closure	6	6
